# Systematic investigation of gastrointestinal diseases in China (SILC): validation of survey methodology

**DOI:** 10.1186/1471-230X-9-86

**Published:** 2009-11-19

**Authors:** Xiaoyan Yan, Rui Wang, Yanfang Zhao, Xiuqiang Ma, Jiqian Fang, Hong Yan, Xiaoping Kang, Ping Yin, Yuantao Hao, Qiang Li, John Dent, Joseph Sung, Duowu Zou, Saga Johansson, Katarina Halling, Wenbin Liu, Jia He

**Affiliations:** 1 Department of Health Statistics, Second Military Medical University, Shanghai, China; 2 Department of Health Statistics, Zhongshan Medical University, Guangzhou, China; 3 Department of Health Statistics, Xi'an Jiao Tong University, Xi'an, China; 4 Department of Health Statistics, Peking University, Beijing, China; 5 Department of Health Statistics, Huazhong Science and Technology University, Wuhan, China; 6 Department of Gastroenterology, Hepatology & General Medicine, Royal Adelaide Hospital, Adelaide, SA, Australia; 7 Department of Medicine and Therapeutics, Chinese University of Hong Kong, Hong Kong, China; 8 Department of Gastroenterology, Changhai Hospital, Second Military Medical University, Shanghai, China; 9 AstraZeneca R&D, Mölndal, Sweden; 10 R&D Medical Affairs, AstraZeneca Pharmaceutical Co Ltd., Shanghai, China

## Abstract

**Background:**

Symptom-based surveys suggest that the prevalence of gastrointestinal diseases is lower in China than in Western countries. The aim of this study was to validate a methodology for the epidemiological investigation of gastrointestinal symptoms and endoscopic findings in China.

**Methods:**

A randomized, stratified, multi-stage sampling methodology was used to select 18 000 adults aged 18-80 years from Shanghai, Beijing, Xi'an, Wuhan and Guangzhou. Participants from Shanghai were invited to provide blood samples and undergo upper gastrointestinal endoscopy. All participants completed Chinese versions of the Reflux Disease Questionnaire (RDQ) and the modified Rome II questionnaire; 20% were also invited to complete the 36-item Short Form Health Survey (SF-36) and Epworth Sleepiness Scale (ESS). The psychometric properties of the questionnaires were evaluated statistically.

**Results:**

The study was completed by 16 091 individuals (response rate: 89.4%), with 3219 (89.4% of those invited) completing the SF-36 and ESS. All 3153 participants in Shanghai provided blood samples and 1030 (32.7%) underwent endoscopy. Cronbach's alpha coefficients were 0.89, 0.89, 0.80 and 0.91, respectively, for the RDQ, modified Rome II questionnaire, ESS and SF-36, supporting internal consistency. Factor analysis supported construct validity of all questionnaire dimensions except SF-36 psychosocial dimensions.

**Conclusion:**

This population-based study has great potential to characterize the relationship between gastrointestinal symptoms and endoscopic findings in China.

## Background

The epidemiology of common gastrointestinal diseases differs between populations in Asian and in Western countries, and in Asia particularly, the pattern of these diseases seems to be changing [1]. The prevalence of peptic ulcer disease has been declining at the same time as the prevalence of gastroesophageal reflux disease (GERD) and its complications have been increasing [2]. Gastric cancer remains a common cancer in Asia, but its prevalence has also been declining in recent years [1]. These changes may reflect the experience of Western countries several decades ago, and comparisons of the epidemiology of gastrointestinal diseases between Western and Asian countries continue to be of interest.

GERD is a chronic disease in which reflux of gastric contents into the esophagus causes a broad range of troublesome symptoms and esophageal complications. The recent Montreal consensus states that a diagnosis of GERD can be made based on symptoms alone and, in population-based surveys, a defined symptom threshold is required for making a symptom-based diagnosis [3]. The historical lack of a single definition of GERD makes comparisons between studies difficult. A systematic review of studies that defined GERD as symptoms of heartburn and/or acid regurgitation experienced at least once a week concluded that GERD is relatively uncommon in Asia, with a prevalence of approximately 5%; this compared with 10-20% in Western countries [4]. However, the recommendations of the Montreal consensus [3] have not yet been applied to epidemiological studies of GERD. Furthermore, the existence of essentially asymptomatic reflux esophagitis - seen in studies of Asian individuals undergoing upper gastrointestinal endoscopy as part of a routine health check [5,6] and population-based endoscopic studies of GERD conducted in Europe [7,8] - means that population-based endoscopic studies are needed to understand the epidemiology of GERD in Asia.

Irritable bowel syndrome (IBS) and dyspepsia are both common gastrointestinal disorders in the West, affecting 10-20% [9] and 20-40% [10] of the population, respectively. They are frequently diagnosed based on symptoms, and often coexist in the same patient [11]. Data on the epidemiology of IBS and dyspepsia are limited in Asian countries, and prevalence estimates vary widely [12,13]. However, the prevalence of IBS appears to be lower in Asia than in the West [14], with a prevalence of 3.8-6.6% in China according to Rome II criteria [15-18]. The prevalence of dyspepsia in populations from Korea, Taiwan and China is 11.7-18.4%, according to Rome II criteria [13,19]. Further studies of IBS, dyspepsia and their relationship to each other, and to GERD, are needed in Asia.

The variation in prevalence of *Helicobacter pylori *infection is one potential explanation for the differences in the epidemiology of upper gastrointestinal diseases between Asian and Western populations and the changing patterns seen in Asia [2]. *H. pylori *infection remains more prevalent in Asia than in the West [20,21], where infection rates have declined in recent decades alongside improving socioeconomic conditions and the availability of eradication therapy [21,22]. Eradication of *H. pylori *has an important role in the treatment or prevention of peptic ulcer disease, dyspepsia and gastric cancer [21,23]. However, the role of *H. pylori *in GERD remains controversial [24]; *H. pylori *infection has been negatively associated with GERD in population studies from Asia, but the results of eradication studies in patients with peptic ulcer disease are inconsistent [25,26].

Asian population-based studies, including endoscopic data and testing for *H. pylori*, are required to clarify the complex relationships between symptom-based GERD, reflux esophagitis, dyspepsia, IBS, peptic ulcer disease and *H. pylori *infection. We have previously conducted a pilot study in Shanghai to validate the methodology for the epidemiological study of GERD in China [27]. This article reports the validation of a refined methodology in the Systematic Investigation of Gastrointestinal Diseases in China (SILC) study, which set out to investigate the epidemiology of upper and lower gastrointestinal diseases in five regions across China using symptom questionnaires that have undergone appropriate validation. The study aimed to collect reliable information on symptom patterns, comorbidities, health-related quality of life and findings from endoscopy, esophageal biopsy and laboratory investigations, including tests for *H. pylori *infection.

## Methods

### Setting

**Figure 1:**
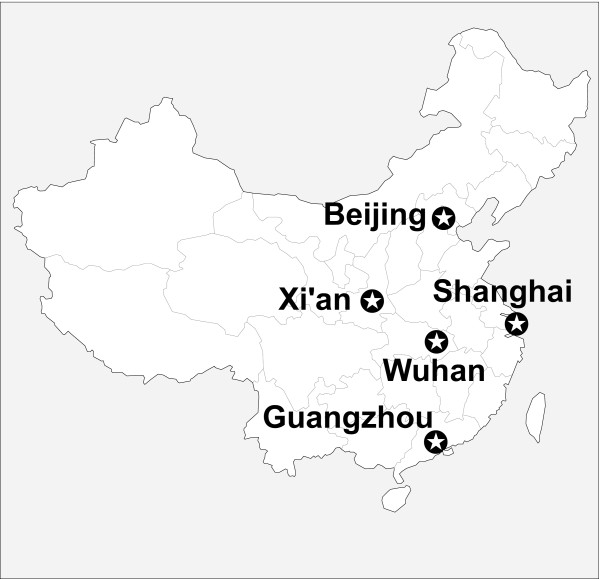
**The five study regions in China**.

This study was conducted in five diverse regions of China, all of which are major population centres (Figure 1).

### Sampling

In total, 18 000 adults aged 18-80 years were randomly selected from Shanghai, Beijing, Xi'an, Wuhan and Guangzhou, using a stratified, multi-stage sampling methodology. Given that urban and rural populations are widely divergent in terms of environment and socioeconomic status, and because approximately half of the residents of these centres live in rural areas (Shanghai: 54.2%; Beijing: 42.5%; Xi'an: 50.4%; Wuhan: 41.4%; Guangzhou: 55.7% [28-33]), urban and rural populations were treated as two separate strata and sampled in a ratio of 1:1 (n = 1800 for each stratum in each region). At the first sampling stage, one or more districts from the urban stratum and one or more counties from the rural stratum were randomly selected from each region. At the second stage, one or more blocks were randomly selected from the urban districts, and one or more townships from the rural counties. At the third stage, one or more residential areas were randomly sampled from the urban blocks, and one or more villages from the rural townships.

All residents of the selected residential areas or villages were stratified according to their age and sex, and individuals were randomly selected from these strata in proportion to the overall age and sex distribution of the population in that region, using data from recent government population surveys (*The Fifth Population Census In China*, 2000, or the 1% sample survey, 2005) [34-39]. Residents who were younger than 18 years, older than 80 years, illiterate, or who had major psychiatric illness or severe visual, hearing or learning disabilities, were excluded from the study.

### Study design

**Figure 2:**
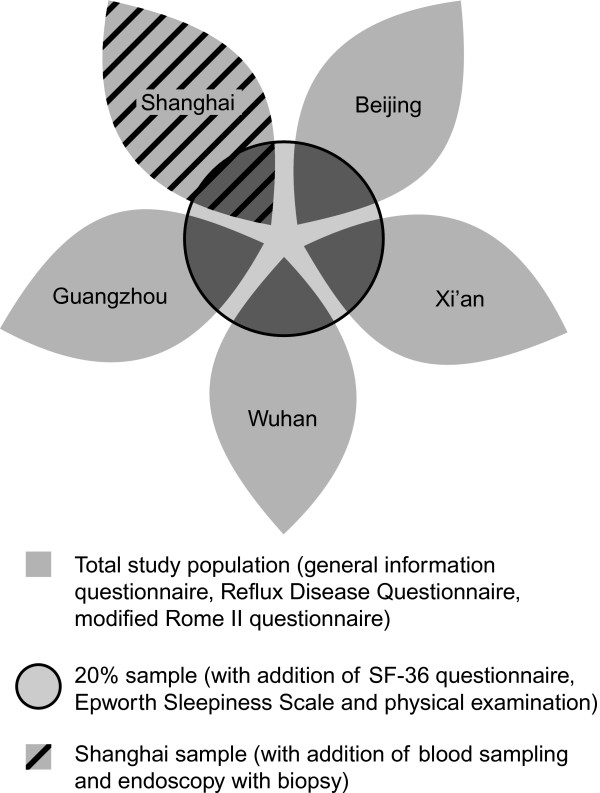
**The survey sampling design**.

All selected individuals were asked to complete a general information questionnaire, and Chinese versions of the Reflux Disease Questionnaire (RDQ) [40] and modified Rome II questionnaire (Figure 2) [41]. In addition, a random sub-sample of the total sample, 20% in each region, was asked to complete Chinese versions of the 36-item Short-Form Health Survey (SF-36) [42] and the Epworth Sleepiness Scale (ESS) [43] and to undergo a physical examination that included measurement of weight (kg), height (cm), and waist and hip circumference (cm). Measurements were made with individuals wearing indoor clothing without shoes. Weight was measured on an electronic scale to the nearest 0.1 kg, and height and circumference measurements were recorded to the nearest centimetre.

All participants from the Shanghai region were invited to undergo upper gastrointestinal endoscopy with esophageal biopsy. Endoscopic results were classified using the Los Angeles (LA) classification of reflux esophagitis [44] and the Prague C and M classification of endoscopically suspected esophageal metaplasia [45]. Markers of microscopic esophagitis from biopsies were classified using criteria developed by an international panel of expert pathologists [46]. Individuals in Shanghai were also asked to provide blood samples for *H. pylori* antibody serology using an immunoglobulin G enzyme-linked immunosorbent assay (ELISA) (Biohit, Helsinki, Finland).

### Administration

The fieldwork period was from April 2007 to January 2008. Questionnaires were self-administered, with trained and supervised facilitators available to explain any questions that respondents found unclear. (Most queries were about the questionnaire formats, such as the skip rules in the modified Rome II questionnaire.) Participants completed the questionnaires at home or in local residential committee offices. Three attempts were made to contact a resident before he or she was considered to be a non-responder.

To encourage high response rates, local residential committee staff publicized the survey in the selected residential areas and villages using leaflets and posters. They also supported participation by setting up early morning and weekend sessions in local residential committee offices, where individuals could come to complete the questionnaires without this interfering with their daily routines. In addition, a small gift (such as shampoo or washing powder) was offered to reward participation. In the Shanghai region, the diagnostic benefits of a free blood test and endoscopy with biopsy were explained, along with the need to fast overnight. Breakfast was provided for those who underwent blood tests and/or endoscopy, and participants were transported to and from the hospital where endoscopy was performed. Individuals were subsequently provided with all test results.

The study was staffed by graduates from the Department of Health Statistics, Second Military Medical University in Shanghai, who received training centrally from gastrointestinal specialists and epidemiologists in Shanghai. Most had also taken part in the pilot survey in Shanghai and were familiar with the survey process. These study staff provided standardized training for facilitators, who were local university graduates or social workers at the sampled sites, before fieldwork commenced. After each questionnaire had been completed, it was checked for completeness and signed by the responsible member of the study staff before the participant received their gift.

Informed consent was obtained from study participants, who were free to discontinue their involvement in the study at any time. The study was approved by the Ethics Committee of the Second Military Medical University in Shanghai, China.

### Questionnaires

The general information questionnaire collected data on age, height, weight, sex, marital status, education, income, occupation, lifestyle, self-reported health status, family history of gastrointestinal diseases, and medical history (current and previous medical problems and related treatment). Current smoking was defined as smoking at least one cigarette daily, and drinking alcohol was classified as alcohol consumption on four or more occasions per month. Current occupation was classified as white-collar worker (including government employees, professionals and technicians), manufacturing industry worker, agricultural or fisheries worker, and other (including service sector and students). Participants indicated their health status, total monthly family income and weekly level of recreational exercise by selecting an option from appropriate categories.

The RDQ is a 12-item, patient-reported questionnaire that assesses the frequency and severity of upper gastrointestinal symptoms such as heartburn, regurgitation and epigastric pain. In this study, a 1-month recall period was used. Each item was scored on a 6-point Likert scale (Table 1). Traditionally, the items are combined into the dimensions of heartburn (burning behind the breastbone and pain behind the breastbone), regurgitation (acid taste in the mouth and movement of materials upwards from the stomach) and epigastric pain (epigastric pain and epigastric burning) [40]. A GERD dimension can be obtained by combining the heartburn and regurgitation dimensions, and the epigastric pain dimension is also known as the dyspepsia dimension. There is strong evidence supporting the validity and reliability of the RDQ as a diagnostic tool in primary care [47] and as a measure of treatment response in clinical trials [48,49]. Previous investigators found that a Chinese version of the RDQ tested in 10 hospitals in China was able to identify the presence of reflux symptoms experienced during the previous month [50]. The Chinese version of the RDQ used in the present study underwent extensive linguistic validation, including forward and backward translation, cognitive debriefing of patients with GERD, and expert input from gastroenterologists. The pilot study in Shanghai showed that a version with a 1-week recall period had credible reliability and construct validity [27]. In the current study, an item was added immediately after the RDQ that asked participants to rate how troublesome they found the symptoms that they had scored in the RDQ overall, using a 5-point scale (where the lowest score was 'not at all' and the highest score was 'extremely').

**Table 1:** Scoring system of the Reflux Disease Questionnaire (RDQ).

Item	Frequency					
	
	None	Less than one day a week	One day a week	2-3 days a week	4-6 days a week	Daily
Burning behind the breastbone	0	1	2	3	4	5
Pain behind the breastbone	0	1	2	3	4	5
Acid taste in the mouth	0	1	2	3	4	5
						
Unpleasant movement of material upwards from the stomach	0	1	2	3	4	5
Epigastric burning	0	1	2	3	4	5
						
Epigastric pain	0	1	2	3	4	5

**Item**	**Severity**					
	
	**None**	**Very mild**	**Mild**	**Moderate**	**Moderately severe**	**Severe**

Burning behind the breastbone	0	1	2	3	4	5
Pain behind the breastbone	0	1	2	3	4	5
Acid taste in the mouth	0	1	2	3	4	5
						
Unpleasant movement of material upwards from the stomach	0	1	2	3	4	5
Epigastric burning	0	1	2	3	4	5
						
Epigastric pain	0	1	2	3	4	5

The Rome II questionnaire is designed to assess symptoms of upper and lower functional gastrointestinal disorders, including esophageal disorders, gastroduodenal disorders, bowel disorders, functional abdominal pain, biliary disorders and anorectal disorders, in a 3-month recall period [41]. The Rome II questionnaire is widely used in research and clinical practice, and there is evidence supporting its validity across various cultures in Asia and the West, including a Chinese version in China [51-53]. The version of the Rome II questionnaire used in this study was translated into Chinese through a process of forward- and back-translation and reconciliation, and then tested for linguistic validity through a process of cognitive debriefing with literate volunteers. It was also modified by removal of reflux items covered by the RDQ, to shorten the survey for participants and minimize repetition and possible confusion. Individuals answered yes or no to eight mandatory items, and a further 16 items covering gastroduodenal, bowel and biliary symptoms were completed only if relevant.

The SF-36 is a generic questionnaire that is widely used to assess health status and well-being during the previous 4 weeks. It contains 36 items, clustered into eight dimensions (physical functioning, role-physical, bodily pain, general health, vitality, social functioning, role-emotional and mental health), plus one item on health change during the previous year [42]. The score for each dimension is the sum of the scores of each item it contains, transformed to a value on a scale of 0 to 100, with high scores indicating good health-related quality of life. The reliability and validity of the SF-36 are well documented in a range of language versions, including Chinese [54-56]. The psychometric properties of the Chinese version of the SF-36 used in this study were assessed during the pilot study in Shanghai [27]. The questionnaire was found to have credible reliability and construct validity.

The ESS is an eight-item, self-administered questionnaire that is widely used to measure daytime sleepiness in adults [43]. The likelihood of dozing in various everyday situations is scored on a 4-point Likert scale, where a score of 3 indicates a high risk of dozing during the daytime and a score of 0 indicates no risk of dozing. Item scores are summed to produce a final score in the range 0-24. ESS scores above 12 suggest problems with excessive sleepiness, scores of 10-12 are borderline, and scores below 10 are considered normal. The reliability of the ESS has been demonstrated in English in Australia [57] and in Chinese in Hong Kong [58]. For use in the present study, it was translated into Chinese through a process of forward- and back-translation and reconciliation, and then tested for linguistic validity through a process of cognitive debriefing with literate volunteers.

### Data collection and analysis

Coding and double entry of questionnaire responses were carried out by two independent professional data-entry staff from the Department of Health Statistics. EpiData software (EpiData Foundation, Odense, Denmark) [59] was used to check for consistency between the two sets of data entries to ensure data quality. SAS 9.1.3 software (SAS Institute, Cary, NC, USA) was used for the data analyses. The mean value of the completed items was used to impute missing values where at least 50% of the items in an RDQ or SF-36 dimension or in the overall ESS had been completed. When values were missing for more than 50% of items, the dimension score (or questionnaire score for the ESS) was excluded from the analysis [60-62]. For the modified Rome II questionnaire, imputation was not performed if an item score was missing.

The internal consistency of survey instruments was evaluated using Cronbach's alpha coefficient to determine the extent to which items within each questionnaire were interrelated [63]. Cronbach's alpha coefficients for each questionnaire were calculated by correlating all individual item scores with dimension scores and/or the overall score. An alpha coefficient above 0.70 suggested good internal consistency. Correlation and/or factor analyses were used to evaluate the construct validity of the RDQ, Rome II questionnaire and the SF-36 - in other words, whether each questionnaire actually measures the phenomena that it theoretically predicts. Factor analysis using principal component analysis and quartimax rotation was employed to explore the factor structure of each questionnaire. Factor loadings larger than 0.50 within one dimension were considered to support the factor construct, provided that the factor loadings were low across the other dimensions; cumulative rates were used to show the contributions of combinations of principal components [64]. Correlation analysis was used to measure the strength of association between dimension scores and the total score for the SF-36 questionnaire, and between item scores and dimension scores for the RDQ. A correlation coefficient of more than 0.6 was considered to indicate a strong correlation, 0.3-0.6 a moderate correlation, and less than 0.3 a weak correlation [65].

Body mass index (BMI) was calculated from self-reported height and weight measurements from the general information questionnaire and, in the 20% sub-sample, from measurements taken by study staff as part of the physical examination. Respondents were categorized on the basis of BMI as having a low risk of cardiovascular disease and type 2 diabetes (< 18.5 kg/m^2^, underweight), an increasing but acceptable risk (18.5-22.9 kg/m^2^, normal weight), an increased risk (23.0-27.4 kg/m^2^, overweight) or a high risk (≥ 27.5 kg/m^2^, obese) [66]. Agreement between participant-reported height and weight and the measurements made by study staff was assessed using the intraclass correlation coefficient (ICC).

### Disease definitions

Based on the Montreal definition of GERD for population-based studies [3], the RDQ was used to define symptom-defined GERD as mild symptoms of heartburn ('burning behind the breastbone' and/or 'pain behind the breastbone') and/or regurgitation ('acid taste in the mouth' and/or 'unpleasant movement of materials upwards from the stomach') occurring on at least 2 days a week (RDQ item frequency score ≥ 3 for a severity score of ≥ 2), or moderate/severe symptoms of heartburn and/or regurgitation occurring on at least 1 day a week (RDQ item frequency score ≥ 2 for a severity score ≥ 3).

As described above, reflux esophagitis was graded using the LA classification system [44] and endoscopically suspected esophageal metaplasia (suspected Barrett's esophagus) was assessed using the Prague C and M criteria [45]. Hiatal hernia was defined as gastric folds extending at least 2 cm above the diaphragmatic hiatus during quiet respiration.

Peptic ulcer disease was defined as a defect in the gastric or duodenal wall that extended through the muscularis mucosae into the deeper layers of the wall (submucosa or the muscularis propria). *H. pylori *test results were classified as positive (≥ 38 enzyme immunoassay units [EIU]) or negative (< 38 EIU) [67].

Atrophic gastritis was defined on the basis of the more severely affected of the serum pepsinogen (PG) I concentration and the PGI/PGII ratio. Results were classified according to the manufacturer's recommendations for China. PGI levels < 30 μg/L and/or a PGI/PGII ratio < 3 indicated severe atrophy (achlorhydric or very hypochlorhydric conditions). PGI levels from 30 μg/L to < 50 μg/L and/or a PGI/PGII ratio from 3 to < 5 indicated moderate atrophy. PGI levels from 50 μg/L to < 70 μg/L and/or a PGI/PGII ratio from 5 to < 7 indicated mild atrophy (hypochlorhydric conditions). Levels above the cut-offs but in the presence of *H. pylori *antibodies indicated non-atrophic *H. pylori *gastritis.

IBS was defined according to the Rome II criteria as abdominal discomfort or pain that has at least two of the following three features: (1) relieved with defecation; (2) onset associated with a change in frequency of stool; and/or (3) onset associated with a change in form (appearance) of stool [68]. IBS was described as diarrhoea-predominant (IBS-D) if patients had one or more of the following: more than three bowel movements a day, loose (mushy) or watery stools, and/or urgency (having to rush to have a bowel movement); and none of the following: fewer than three bowel movements a week, hard or lumpy stools, and/or straining during a bowel movement. IBS was described as constipation-predominant (IBS-C) if patients had one or more of the following: fewer than three bowel movements a week, hard or lumpy stools, and/or straining during a bowel movement; and none of the following: more than three bowel movements a day, loose (mushy) or watery stools, and/or urgency (having to rush to have a bowel movement).

Dyspepsia was defined according to the Rome II criteria as persistent or recurrent pain or discomfort centred in the upper abdomen, with no evidence that symptoms are exclusively relieved by defecation or associated with the onset of a change in stool frequency or stool form [69]. Dyspepsia was described as ulcer-like if the predominant (most bothersome) symptom was pain centred in the upper abdomen. Dyspepsia was described as dysmotility-like if the predominant symptom was an unpleasant or troublesome nonpainful sensation (discomfort) centred in the upper abdomen; this sensation may be characterized by or associated with upper abdominal fullness, early satiety, bloating or nausea.

## Results

### Response rate

In total, 16 091 individuals completed the questionnaires within a period of 10 months in 2007 and 2008. The response rate ranged from 87.6% to 91.4% in the different regions, and the overall response rate was 89.4% (Table 2). In all five regions, the response rate was lower in men (78.7-89.9%) than in women (91.5-98.4%), and was generally lowest in the youngest age group (73.1-85.2% in 18-29 year-olds). Nearly half of the non-responders to the survey were in the 18-29-year-old age group; the response rate in this group was lower in men (75.2%) than in women (86.5%) overall and in all regions (data not shown). Thirteen participants were excluded from the analysis because of logical errors or insufficient completion of questionnaires, leaving a total of 16 078 individuals suitable for inclusion in the analysis.

**Table 2:** Numbers of participants (and response rates) for the randomized samples in each region.

	Shanghai	Beijing	Wuhan	Xi'an	Guangzhou	Total
	
Total sample	3153 (87.6%)^a^	3171 (88.1%)	3291 (91.4%)	3266 (90.7%)	3210 (89.2%)	16 091 (89.4%)
80% sub-sample	2510 (87.2%)^a^	2579 (89.5%)	2615 (90.8%)	2622 (91.0%)	2546 (88.4%)	12 872 (89.4%)
• General information questionnaire						
• RDQ						
• Modified Rome II questionnaire						

20% sub-sample	643 (89.3%)^a^	592 (82.2%)	676 (93.9%)	644 (89.4%)	664 (92.2%)	3219 (89.4%)
• General information questionnaire						
• RDQ						
• Modified Rome II questionnaire						
• SF-36						
• ESS						
• Physical examination						

ESS, Epworth Sleepiness Scale; GI, gastrointestinal; RDQ, Reflux Disease Questionnaire; SF-36, 36-item Short-Form Health Survey.

^a^In Shanghai, all 3153 respondents to the initial survey underwent blood sampling (100% response rate), and 1030 participants (32.7%) accepted the invitation to undergo upper gastrointestinal endoscopy with esophageal biopsy, 764 (30.4%) in the 80% sub-sample and 266 (41.4%) in the 20% sub-sample

In the 20% sub-sample, 3219 individuals responded, equating to a response rate of 89.4% overall (82.2-93.9% in the different regions), and data from 3214 individuals were suitable for analysis (Table 2). In the Shanghai sample, all 3153 participants (100%) provided blood samples, of which 3151 were suitable for inclusion in the analysis, and 1030 (32.7%) volunteered to undergo endoscopy with biopsy, of whom 1029 were suitable for inclusion in the analysis of endoscopic data. Six participants did not undergo biopsy because they met biopsy exclusion criteria (e.g. presence of esophageal varices or angioma), leaving 1022 individuals with biopsy results suitable for analysis.

### Participants

The mean age of the participants was 43 years, and 52% were women (Table 3). BMI ranged from 11.8 kg/m^2 ^to 41.0 kg/m^2^, with a mean BMI of 22.6 kg/m^2^. According to the BMI ranges appropriate for the population [66], 34.4% of participants were overweight and 8.1% were obese. When self-reported height and weight data were compared with measurements taken by study staff in the 20% sub-sample, ICCs of 0.97 and 0.98, respectively, were obtained, indicating that the self-reported values were reliable. Most participants were married (78%), did not smoke (72%) and did not drink alcohol (80%). Almost all smokers (95.6%) and drinkers (92.8%) were male. Further socioeconomic characteristics of participants are summarized in Table 4. The age and sex distribution of the participants was broadly representative of the distribution of the general population in each region (Table 5), although individuals in the youngest age groups were slightly under-represented at each study site.

**Table 3:** Demographics and lifestyle characteristics of participants by region.

Variables	Shanghai (n = 3151)	Beijing (n = 3168)	Wuhan (n = 3283)	Xi'an (n = 3266)	Guangzhou (n = 3210)	Total (n = 16 078)
**Age (years)**	47.7 ± 14.1	42.7 ± 15.3	41.5 ± 15.4	41.6 ± 15.2	39.0 ± 14.3	42.5 ± 15.2
**Weight (kg)**	63.0 ± 11.4	65.1 ± 11.4	60.1 ± 10.1	60.8 ± 10.2	57.8 ± 9.9	61.3 ± 10.9
**Height (cm)**	164.4 ± 8.1	165.8 ± 8.0	164.1 ± 7.6	165.3 ± 7.7	162.4 ± 8.1	164.4 ± 8.0
**Body mass index (kg/m^2^)**	23.2 ± 3.3	23.7 ± 3.5	22.3 ± 3.2	22.2 ± 3.2	21.9 ± 3.1	22.6 ± 3.3
**Female sex**	1749 (55.5)	1682 (53.1)	1719 (52.4)	1647 (50.4)	1593 (49.6)	8390 (52.2)
**Urban area**	1572 (49.9)	1551 (49.0)	1653 (50.4)	1617 (49.5)	1679 (52.3)	8072 (50.2)
**Smoking**	926 (29.4)	962 (30.4)	899 (27.4)	912 (27.9)	732 (22.8)	4431 (27.6)
**Alcohol use (≥ 4 times per month)**	628 (19.9)	780 (24.6)	791 (24.1)	528 (16.2)	535 (16.7)	3262 (20.3)
**Family history of gastrointestinal disease**	487 (15.5)	98 (3.1)	396 (12.1)	275 (8.4)	171 (5.3)	1427 (8.9)
**Married**	2693 (85.5)	2421 (76.4)	2597 (79.1)	2583 (79.1)	2288 (71.3)	12 582 (78.3)

Data are mean ± standard deviation or number (%) of participants.

**Table 4:** Socioeconomic characteristics of participants by region.

Variables	Shanghai (n = 3151)	Beijing (n = 3168)	Wuhan (n = 3283)	Xi'an (n = 3266)	Guangzhou (n = 3210)	Total (n = 16 078)
**Maximum education level**						
Primary school/uneducated	595 (18.9)	427 (13.5)	914 (27.8)	659 (20.2)	587 (18.3)	3182 (19.8)
Secondary/high school	2162 (68.6)	1884 (59.5)	2056 (62.6)	1920 (58.8)	1908 (59.4)	9930 (61.8)
College graduate	394 (12.5)	857 (27.1)	312 (9.5)	687 (21.0)	714 (22.2)	2964 (18.4)
**Current occupation^a^**						
White-collar worker	756 (24.0)	666 (21.0)	395 (12.0)	844 (25.8)	897 (27.9)	3558 (22.1)
Manufacturing industry worker	680 (21.6)	299 (9.4)	464 (14.1)	297 (9.1)	739 (23.0)	2479 (15.4)
Agricultural or fisheries worker	729 (23.1)	1343 (42.4)	1083 (33.0)	1355 (41.5)	302 (9.4)	4812 (29.9)
Other	985 (31.3)	858 (27.1)	1333 (40.6)	766 (23.5)	1266 (39.4)	5208 (32.4)
**Total family income per month^b^**						
≤ 1999 yuan	1246 (39.5)	1727 (54.5)	2132 (64.9)	2236 (68.5)	1474 (45.9)	8815 (54.8)
2000-4999 yuan	1574 (50.0)	1240 (39.1)	989 (30.1)	899 (27.5)	1261 (39.3)	5963 (37.1)
≥ 5000 yuan	324 (10.3)	192 (6.1)	160 (4.9)	130 (4.0)	453 (14.1)	1259 (7.8)

Data are number (%) of participants.

^a^White-collar workers included government employees, technicians and professionals, and other workers included service sector employees and students.

^b^Average yearly income in China in 2007: 24 932 yuan (~$3300) [76].

NOTE: numbers may not add up exactly to 100% for each socioeconomic category where individual participants have refused to answer specific questions on the general information questionnaire.

**Table 5:** Age and sex distribution in the general population and in study participants in each region.

	Shanghai (%)		Beijing (%)		Wuhan (%)		Xi'an (%)		Guangzhou (%)	
	**Population^a^** **(n = 13.5 m)**	**Participants** **(n = 3151)**	**Population^b^** **(n = 11.8 m)**	**Participants** **(n = 3168)**	**Population^b^** **(n = 7.8 m)**	**Participants** **(n = 3283)**	**Population^a^** **(n = 7.3 m)**	**Participants** **(n = 3266)**	**Population^b^** **(n = 7.3 m)**	**Participants** **(n = 3210)**

**Female**	50.6	55.5	50.0	53.1	49.7	52.4	50.0	50.4	45.4	49.6

**Urban**	54.2	49.9	57.5	49.0	58.6	50.4	50.4	49.5	55.7	52.3

**Age (years)**										

**18-29**	13.3	11.1	25.6	23.9	31.1	26.5	27.8	24.8	31.9	27.8

**30-39**	18.8	15.8	21.5	21.1	19.6	22.8	25.7	25.2	28.5	29.2

**40-49**	30.2	33.8	22.0	23.1	20.3	20.8	19.9	21.2	17.8	19.9

**50-59**	16.4	18.9	15.1	16.0	15.5	15.4	12.9	13.8	11.5	12.7

**60-69**	12.4	12.1	9.3	9.8	8.0	8.5	8.8	9.5	6.3	7.0

**70-80**	9.1	8.3	6.5	6.1	5.5	6.1	5.0	5.4	4.0	3.5

^a^Data from the fifth population census in China (2000) [35,39].

^b^Data from the government 1% sample survey (2005) [36-38].

m, million.

Several demographic variables differed between those who did and those who did not volunteer for endoscopy in the Shanghai region. Greater proportions of participants who underwent endoscopy lived in a rural environment (63.1%, versus 43.8% of those who did not undergo endoscopy) or had a family history of gastrointestinal diseases (23.7% versus 11.5%), whereas the proportions of individuals who were in the youngest age range (18-29 years; 5.3% versus 13.9%), who were unmarried (3.8% versus 11.9%) or who were college graduates (8.9% versus 14.2%) were lower among the population who underwent endoscopy than the population that did not (all *p *≤ 0.001). Sex, BMI, occupation, annual income, smoking status and alcohol consumption were similar among those who underwent endoscopy and those who did not.

### Reliability

Internal consistency (indicated by Cronbach's alpha coefficient) was above 0.7 for all questionnaires, demonstrating good reliability. Cronbach's alpha coefficient was 0.89 for the RDQ, 0.89 for the modified Rome II questionnaire, 0.80 for the ESS, and 0.91 for the overall SF-36. For seven of the eight individual SF-36 dimensions, Cronbach's alpha coefficient was above 0.7 (0.75-0.92), but for social functioning it was 0.51.

### Construct validity

The RDQ, modified Rome II and SF-36 all demonstrated credible construct validity using correlation and/or factor analyses. For the RDQ, each dimension correlated most strongly with the items comprising it (Spearman correlation coefficients 0.63-0.87, *p *< 0.001; Table 6). Factor analysis of the RDQ indicated that the 12 items distributed to four factors that supported the theoretical construct of the RDQ (one representing the regurgitation dimension, another representing the heartburn dimension and two representing the dyspepsia dimension). Factor loadings ranged from 0.67 to 0.88, and the cumulative rate of the four factors was 80.1%.

**Table 6:** Correlation between Reflux Disease Questionnaire (RDQ) item score and dimension scores (correlation coefficients ≥ 0.6 are in bold).

RDQ item	Spearman correlation coefficient			
	
	Regurgitation dimension	Heartburn dimension	GERD dimension	Dyspepsia dimension
**Burning behind breastbone**				
Frequency	0.37	**0.84**	**0.67**	0.42
Severity	0.37	**0.84**	**0.66**	0.42
**Pain behind breastbone**				
Frequency	0.31	**0.85**	**0.63**	0.40
Severity	0.32	**0.86**	**0.64**	0.41
**Acid taste**				
Frequency	**0.85**	0.34	**0.77**	0.40
Severity	**0.87**	0.32	**0.77**	0.40
**Movement of materials**				
Frequency	**0.80**	0.34	**0.72**	0.38
Severity	**0.81**	0.33	**0.73**	0.38
**Epigastric burning**				
Frequency	0.42	0.46	0.52	**0.78**
Severity	0.40	0.44	0.50	**0.78**
**Epigastric pain**				
Frequency	0.36	0.37	0.44	**0.86**
Severity	0.37	0.34	0.42	**0.85**

GERD, gastroesophageal reflux disease.

Factor analysis of the modified Rome II questionnaire revealed that the 37 items distributed to seven factors with factor loadings that ranged from 0.57 to 0.94. The cumulative rate of the seven factors was 78.9%. These factors, representing dimensions of functional dyspepsia, IBS, an IBS subtype, biliary disorders, functional vomiting, aerophagia and an integrated dimension, provide empirical support for the validity of the Rome II classification system in China.

For the SF-36, all dimensions correlated most strongly with the items comprising it, except the physical functioning dimension. Spearman correlation coefficients for the association between an item score and its dimension score were greater than 0.6 (range 0.61-0.99) for all items except seven out of the ten physical functioning items (range 0.25-0.50). In factor analysis of the SF-36, the 36 items distributed to eight factors that generally supported the theoretical construct of the questionnaire (cumulative rate of 65.5%). Most items were distributed as expected from the theoretical construct (factor loadings 0.48-0.86), except for all mental health and vitality items, which were distributed to two factors (factor loadings 0.61-0.78); the social functioning items, which did not resolve into a distinct factor, but instead were distributed most strongly to role-emotional and mental health/vitality factors (factor loadings 0.45 and 0.43, respectively); and one general health item, which was also not clearly distributed to the expected factor.

## Discussion

The purpose of this study was to evaluate the validity of the methodology used to conduct the largest multicentre population-based survey of gastrointestinal symptoms in China to date, and the first to include upper gastrointestinal endoscopy with biopsy. The methodology was based on that applied in a pilot study in Shanghai [27], which verified the feasibility of conducting this larger and more extensive multicentre study.

High response rates were achieved to the simple, validated questionnaires used and, although the youngest age group was slightly under-represented, the survey sample was broadly representative of the general population in the survey regions. However, response rates are likely to have been low among migrant workers, who are registered at their place of origin rather than where they have moved in search of work, and who make up more than 10% of the Chinese population [70]. Response rates among individuals invited for physical examination and blood sampling were high, demonstrating that the traditional reluctance of Chinese individuals to provide blood samples can be overcome. As anticipated, the response rate among those invited to undergo endoscopy was lower, and led to some response bias, but the number of participants undergoing endoscopy was still substantial (n = 1030).

The reliability and validity of the survey instruments in the current study were demonstrated for the modified Rome II questionnaire and ESS, and confirmed for the SF-36 and RDQ. Each questionnaire had a high Cronbach's alpha coefficient (≥ 0.8), suggesting good reliability. The internal consistency of the RDQ dimensions replicated findings from the original RDQ, as well as other language versions [48,71]. Although test-retest reliability, known-groups validity and responsiveness to change were not assessed, the test-retest reliability of the Chinese versions of the RDQ and SF-36 was previously established in the Shanghai pilot study [27]. The construct validity of the questionnaires was also credible. As found in the Shanghai pilot study and previous studies in China, the social functioning dimension of the SF-36 tends to perform less well than other dimensions [27,54], and vitality is more strongly associated with mental health among Chinese-speaking people [54,72-75]. It seems likely that these variations reflect underlying cultural perceptions and social standards regarding the relationship between health, energy (or 'qi') and social functioning in China [54]. This limits the reliability of conclusions reached on the basis of data from this SF-36 dimension in China, and highlights the importance of assessing the psychometric validity of survey instruments in different cultures and settings.

## Conclusion

In summary, the methodology used to conduct this large, multicentre epidemiological study of gastrointestinal diseases in five regions across China was valid and credible, and the survey questionnaires demonstrated acceptable psychometric properties. The prevalence of gastrointestinal diseases in these diverse regions of China, together with any associations of symptoms with demographic characteristics, health-related quality of life, sleep disturbance, and endoscopic, biopsy and laboratory findings, will be described in subsequent publications. The SILC study has the potential to make a major contribution to the epidemiological understanding of symptoms of gastrointestinal diseases in China, and to provide insight into the relationship between gastrointestinal symptoms and endoscopic findings that is likely to be of global significance.

## Competing interests

X. Yan, R. Wang, Y. Zhao, X. Ma, J. Fang, H. Yan, X. Kang, P. Yin, Y. Hao, Q. Li and D. Zou declare that they have no competing interests. J. He has served as the Director of the Department of Health Statistics, Second Military Medical University and has received research funding from AstraZeneca. J. Dent has served as a speaker, a consultant and an advisory board member for AstraZeneca, and has received research funding from AstraZeneca. J. Sung has served as a speaker, a consultant and an advisory board member for AstraZeneca, and has received research funding from AstraZeneca. S. Johansson is an employee of AstraZeneca. K. Halling and W. Liu were employees of AstraZeneca at the time the study was conducted; K. Halling is currently employed by PRO Consulting and W. Liu by Genzyme. The study was funded by AstraZeneca R&D, Mölndal, Sweden. AstraZeneca had no role to play in the content and conduct of the study. Writing support was provided by C. Winchester of Oxford PharmaGenesis™ Ltd and funded by AstraZeneca.

## Authors' contributions

XY, RW, YZ, SJ, KH, WL, JH, JD and JS made substantial contributions to the conception and design of the study, XY, RW, YZ, XM, JF, HY, XK, PY, QL, YH, DZ, WL and JH participated in data collection, and XY, RW, YZ, SJ, KH, JH, JD and JS analysed and interpreted the data. All authors have been involved in critically revising the manuscript for intellectual content, and have given final approval of the version to be published.

## Pre-publication history

The pre-publication history for this paper can be accessed here:

http://www.biomedcentral.com/1471-230X/9/86/prepub
